# Effective Interactions between Multilayered Ionic Microgels

**DOI:** 10.3390/ma7127689

**Published:** 2014-12-02

**Authors:** Clemens Hanel, Christos N. Likos, Ronald Blaak

**Affiliations:** Faculty of Physics, University of Vienna, Boltzmanngasse 5, A-1090 Vienna, Austria; E-Mails: clemens.hanel@univie.ac.at (C.H.); ronald.blaak@univie.ac.at (R.B.)

**Keywords:** mutilayered microgels, polyelectrolytes, effective interactions, linear response

## Abstract

Using a one-component reduction formalism, we calculate the effective interactions and the counterion density profiles for microgels that feature a multilayered shell structure. We follow a strategy that involves second order perturbation theory and obtain analytical expressions for the effective interactions by modeling the layers of the particles as linear superpostion of homogeneously charged spheres. The general method is applied to the important case of core–shell microgels and compared with the well-known results for a microgel that can be approximated by a macroscopic, and homogeneously charged, spherical macroion.

## 1. Introduction

Microgels can be described as a colloidal suspension of gel particles, where the individual particles themselves are very large compared with the atomic scale, but rather small compared with the macroscopic level. The typical size range of such microgel particles is about 10–1000 nm and could for instance be formed by polyelectrolyte complex formation when the individual particles consist of a number of shorter polymer chains [1,2,3]. Dispersing microgel particles in a solvent causes them to swell by absorbing the solvent, which can result in doubling their initial radius. One of the most important properties of microgels is that this swelling is reversible and can depend on, and be controlled by, various different external parameters such as the pH value of the solvent, the temperature and the salt concentration. Consequently, this also allows for tuning the pair interaction between gel particles from almost hard sphere-like interactions in the collapsed state to soft repulsions when fully swollen. It should be noted that the swelling does not affect the connectivity within the polymer network, whose stability originates mostly in strong covalent bonding forces.

In recent years, the field of ionic microgels received a considerable amount of attention from theoretical approaches [4,5] as well as experiments [6,7,8,9]. One of the main reasons for this interest is found in the various applications of these systems in industry and medicine. A good illustration of an important application is found in drug delivery methods, where one is interested in using microgels to encapsulate and release pharmaceuticals in a controlled fashion by means of changing one or more external parameters [7,10]. Other applications of microgels include sensor technology [11,12], photonic crystals [13,14] and purification techniques [15]. A particular class of microgels are of ionic nature and formed by copolymers (*i.e.*, polymers consisting of at least two monomeric constituents) made of *N*-isopropylacrylamide (NIPAM) or *N*-vinylcaprolactam (VCL) and an ionizable monomer such as acrylic acid [16,17].

In this work, we focus on the electrostatic interactions of multilayered shell microgels, the most prominent example being core–shell microgels, which for practical purposes can be considered to be huge macroions. The internal structure of such a particle is an interconnected polymeric network and depends on the number and strength of cross-linking. Weak cross-linking usually results in a fairly homogeneous distribution of the polymer chains and therefore the corresponding microgel particles could be regarded as homogeneously charged macroions. However, an increased strength of the cross-linking would have resulted in the polymer chains to rearrange in a fashion that resembles a core–shell structure [18]. That is, the charge density of the macroion changes with respect to the distance from the macroions center. Obviously, such an ionic microgel solution is a multi-component mixture of macroions, counterions, solvent and ions thereof. Since a full analytical treatment in terms of pair interactions of these multi-body systems is in general a formidable challenge, the preferred approach is to treat the system at the level of effective interactions. This can be achieved by tracing out all degrees of freedom up to a single component. In doing so, one obtains an equivalent one-component system of so-called pseudoparticles, which are subjected to an effective mutual interaction.

This approach has been applied to the case of colloidal suspensions of non-penetrable charged macroions [19], solutions of star polymers and homogeneously charged microgel particles [20]; and the case of core–shell particles was investigated by means of employing an integral equation method [21]. Here we generalize and extend the linear response approximation to inhomogeneous microgels. To this end, we follow Denton’s approach [19,20] for a theory of effective interactions based on second order perturbation theory to derive the microion density profiles and the effective interaction between inhomogeneous macroions. We will assume that the macroions are completely penetrable by both counterions and other macroions, and that the external parameters and the internal structure of the macroions are known. Moreover, the inhomogeneous structure of the microgel particles is assumed to be spherically symmetric. It is clear that in general microgel particles will not be fully penetrable due to the presence of cross-links. Such a restriction, however, does not apply to for instance polyelectrolyte dendrimers [22] that can be described within the same framework.

This paper is organized in the following way. In Section 2, we describe the microgel model and present the theoretical approach that is used to calculate the effective interactions via linear response theory. This is followed by Section 3, which is dedicated to the analytical results for multilayered shell microgels. In Section 4 the generally applicable method will be applied to core–shell particles and we conclude in Section 5 with a discussion and summary of the main results.

## 2. Model and Theory

We consider the following model of a microgel solution, based on the theory of ionic liquids as can be found in [23]. The system contains, without loss of generality, a number *N*_m_ of negatively charged spherical macroions with radius *a*, mass *M* and overall charge *−Zq*, and a number *N*_c_ of positively charged point-like counterions of charge *zq* and mass *m*. Here *q* ≡ |*e*| denotes the elementary charge and the positive values *Z* and *z* correspond to the valences of the macroions and counterions, respectively. The macroions and counterions are dispersed in an electrolyte solvent of some volume *V*, which we assume can be fully described in an implicit fashion by means of its dielectric constant *ε* and temperature *T*. The added salt concentration is characterized by fixing the number *N*_s_ of positively as well as negatively charged monovalent microions. For reasons of simplicity, both have been chosen to have the same valence *z* = 1 as the counterions. Thus the system consists of *N*_+_ = *N*_c_ + *N*_s_ positive and *N*_−_ = *N*_s_ negative microions, bringing the total number of microions to *N_µ_*= *N*_c_ + 2*N*_s_ with the corresponding total microion density *n_µ_* = *N_µ_/V*.

To ensure an overall charge neutrality of the system, we have the additional requirement that *zn*_c_*−* *Zn*_m_ = 0, where *n*_m_ = *N*_m_*/V* and *n*_c_ = *N*_c_*/V* are the macroion and counterion densities, respectively. We can distinguish three different characteristic regions where counterions can be found: free counterions outside the macroions, counterions trapped within the macroions but sufficiently far away from the polymer chains to be considered unbounded, and counterions closely surrounding the polymer chains. In our model we assume the counterions in the latter region to be effectively bounded by electrostatic interactions and merely renormalize the effective charge of the macroion.

In the remainder of this section, we summarize the basic ingredients that are necessary to arrive at an effective one-component description of macroions only and those required for the approximation by linear response theory. The combination of these allows us to build up a general framework for microgels consisting of an arbitrary number of layers as is detailed in Section 3. The reader interested in two-layer, core–shell microgels may skip both sections and continue with Section 4, where the general formalism is illustrated and results are shown for this particular application.

### 2.1. One-Component Reduction

The first step in coarse graining the full detailed system of our microgel solution is the reduction to an effective one-component system by integrating out all microions. To this end, we will follow closely the approach as was employed by Denton [19,20,24].

We start by considering the full Hamiltonian of our microgel solution, where we denote by {***R***}, {***P***} the collective coordinates and momenta of the macroions. Similarly, {***r****^σ^*} and {***p****^σ^*} describe the collective coordinates and momenta of the microions, where the superscript label *σ* = *±* refers to either the positively or the negatively charged microions.

The full Hamiltonian can now be separated into four contributions: the macroion energy, the microion energy, and two interaction terms between macro- and microions.

(1) *H*({***R***, ***r****^σ^*, ***P***, ***p****^σ^*}) = *H*_m_ + *H_µ_* + *U*_m__+_ + *U*_m−_

Note that the second term contains all microion interactions, whereas the two last contributions explicitly treat the positive and negative microions separately.

If we examine these contributions in more detail, we find that the first term in Equation (1) represents the sum of the kinetic and potential energy of a gas of interacting macroions. Since this obviously depends on the macroion coordinates only, this can be written as:

(2) $$H_{\text{m}}(\{R,P\})=\frac{1}{2M}\sum_{i=1}^{N_{\text{m}}}|P_{i}{|}^{2}+\sum_{i<j}^{N_{\text{m}}}v_{\text{mm}}(|R_{i}-R_{j}|)$$

Here, *v*_mm_(*r*) represents the bare pair potential of two macroions in the implicit screening solvent and separated by a distance *r*. The actual form of *v*_mm_ depends on the internal configuration of the macroion and will be specified later in Section 3. It should be realized that this particular formulation is only valid within the assumed spherical symmetry of the microgel particles, otherwise additional internal degrees of freedom would appear. Effectively, it also means that already some layer of coarse graining has been applied to the microgel by assuming this symmetry.

In a similar fashion, the second term in Equation (1) yields:

(3) $$\begin{array}{l} H_{µ}(\{r^{\sigma },p^{\sigma }\})=\frac{1}{2m}\sum_{\sigma =\pm }\sum_{i=1}^{N_{\sigma }}{\left|p_{i}^{\sigma }\right|}^{2}\\ +\sum_{\sigma =\pm }\sum_{i<j}^{N_{\sigma }}v_{\sigma \sigma }(|r_{i}^{\sigma }-r_{j}^{\sigma }|)\\ +\sum_{i=1}^{N_{+}}\sum_{j=1}^{N_{-}}v_{+-}(|r_{i}^{+}-r_{j}^{-}|) \end{array}$$

and consists of kinetic and potential energy for all the microions. The microion interaction itself is just the Coulomb pair potential for two point particles separated at a distance *r* and hence, under the assumption of identical valences, we find for the different microion pair interactions:

(4) $$v_{++}(r)=v_{--}(r)=-v_{+-}(r)=\frac{1}{\varepsilon }\frac{q^{2}}{r}$$

The two final terms in Equation (1) describe the electrostatic interaction between macro- and microions and can be expressed as:

(5) $$U_{\text{m}\sigma }(\{R,r^{\sigma }\})=\sum_{i=1}^{N_{\text{m}}}\sum_{j=1}^{N_{\sigma }}v_{\text{m}\sigma }(|R_{i}-r_{j}^{\sigma }|)$$

where *σ* = *±* for the either positively or negatively charged microions, and *v*_m_*_σ_*(*r*) is the corresponding macroion–microion pair interaction. If we now introduce the number densities of macroions and both types of microions respectively by:

(6a) $$\rho_{\text{m}}(R)=\sum_{i=1}^{N_{\text{m}}}\delta (R-R_{i})$$

(6b) $$\rho_{\sigma }(r)=\sum_{i=1}^{N_{\sigma }}\delta (r-r_{i}^{\sigma })$$

we can rewrite Equation (5) in a more convenient form:

(7) $$U_{\text{m}\sigma }(\{R,r_{\sigma }\})=\iint \rho_{\text{m}}(R)\rho_{\sigma }(r)\upsilon_{\text{m}\sigma }(|R-r|)d^{3}rd^{3}R$$

Note that also here the pair interaction *v*_m_*_σ_*(*r*) again will depend on the internal configuration of the macroions and will be specified later in Section 3.

Following the approach for simple metals [23,25], we reduce the two-component mixture, formed by macro- and microions, to an equivalent one-component system. The latter will be governed by an effective Hamiltonian *H*_eff_({***R***, ***P***}), which will only depend on the macroion coordinates. This can be achieved by tracing out the microion degrees of freedom. To this end we consider the canonical partition function derived from the microgel Hamiltonian. With the inverse temperature *β* = 1*/_k_*_B_*T* we have:

(8) $$Z={\text{tr}}_{\text{m}}\left[{\text{tr}}_{\sigma }(e^{-\beta H(\{R,P,r^{\sigma },p^{\sigma }\})})\right]$$

where the trace:

(9) $${\text{tr}}_{\alpha }(f)=\frac{1}{h^{3N_{\alpha }}N_{\alpha }!}\iint f(\{r^{\alpha },p^{\alpha }\})d^{3N_{\alpha }}pd^{3N_{\alpha }}r$$

is taken over the macroions or all microions for respectively the subscripts m and ±.

By carrying out the integration over the microion coordinates and momenta, the canonical partition function can be rewritten as:

(10) $$Z={\text{tr}}_{\text{m}}(e^{-\beta H_{\text{eff}}(\{R,P\})})$$

in terms of an effective Hamiltonian *H*_eff_ = *H*_m_ + *F_µ_*, where:

(11) $$F_{µ}=-\frac{1}{\beta }\text{ln}[{\text{tr}}_{\sigma }(e^{-\beta (H_{µ}+U_{\text{m}+}+U_{\text{m}-}+E_{b})})]$$

is the free energy of a non-uniform gas of microions subjected to an external field generated by macroions at the positions {***R***}. Here a uniform compensating background energy *E*_b_ has been introduced [23,24], which appears in the modified Hamiltonian *H*_m_ = *H*_m_ − *E*_b_, and is balanced by its counterpart in the free energy in Equation (11). The reason for introducing this background energy is that the system of microions in the external field of macroions described by the Hamiltonian *H_µ_* + *U*_m__+_ + *U*_m__−_ does not possess a thermodynamic limit, and consequently the free energy in Equation (11) without this term would be ill-defined. Likewise also *H*_m_, which describes the Hamiltonian of a system consisting of interacting macroions only, does not have a thermodynamic limit. The origin of this problem lies in the fact that the systems of either micro- or macroions are not electrically neutral. This problem can easily be lifted by subtracting the energy term stemming from a homogeneous charge density with the same magnitude; in other words, to introduce modified Hamiltonians and interaction terms that give the difference in energy with respect to the static, homogeneously distributed densities of macro- and microions. In doing so, the divergent contributions arising from concentrating a non-zero charge density in a specified volume are eliminated.

In the case of the macroions, this would result in a homogeneous charge density *−Zqn*_m_ that results in an energy contribution:

(12) $$E_{\text{b}}=\frac{1}{2}\iint \frac{{\left(-Zqn_{\text{m}}\right)}^{2}}{\varepsilon |r-r\prime |}d^{3}rd^{3}r\prime \;=\frac{1}{2}Z^{2}N_{\text{m}}n_{\text{m}}{\hat{v}}_{++}\left(0\right)$$

where *v̂*_++_(0) is the *k* → 0 limit of the Fourier transformed pair interaction between elementary charges. In a similar fashion one would find, in the case of a system of microions only, a modified Hamiltonian *H**_µ_* = *H_µ_* *−* *E*_b_ with:

(13) $$\begin{array}{l} E_{\text{b}}=\frac{1}{2}\sum_{\sigma ,\sigma ＇=\pm }\iint \frac{\left(\sigma qn_{\sigma }\;)(\sigma \prime Iqn_{\sigma ＇\;}\right)}{\varepsilon |r-r\prime |}d^{3}rd^{3}r\prime \\ =\frac{1}{2}(N_{+}-N_{-})(n_{+}-n_{-}){\hat{v}}_{++}\left(0\right) \end{array}$$

Obviously these two values have to be the same, since in either case it describes the energy required to concentrate the same magnitude of charge, but opposite signs, within a specified volume, and indeed equality follows directly from the combined system neutrality condition for the densities.

Similar terms are also found for the interaction terms between macro- and microions, which results in the modified interaction contributions:

(14) $$\begin{array}{l} {\overset{-}{U}}_{\text{m}\sigma }=U_{\text{m}\sigma }-\iint \frac{\left(-Zqn_{\text{m}}\;)(\sigma qn_{\sigma }\right)}{\varepsilon |r-r\prime |}d^{3}rd^{3}r\prime \\ =U_{\text{m}\sigma }+\frac{2\sigma n_{\sigma }}{n_{+}-n_{-}}E_{\text{b}} \end{array}$$

and hence the free energy in Equation (11) in terms of the modified Hamiltonians and interactions could also be written as:

(15) $$F_{µ}=-\frac{1}{\beta }{\text{ln[tr}}_{\sigma }(e^{-\beta ({\overset{-}{H}}_{µ}+{\overset{-}{U}}_{\text{m+}}+{\overset{-}{U}}_{m-})})]$$

Note that the volume integrations in Equations (12) and (13), similar to Denton [20], have been chosen to be carried out over the full volume rather than a restricted volume. This corresponds to the implicit assumption that the macroions do not possess an impenetrable hard-core, as was for instance used in [24].

### 2.2. Approximation by Linear Response Theory

For the second ingredient in the description of inhomogeneous microgels, perturbation theory is employed in order to approximate the free energy of the system. This enables us to calculate the microion density profiles and the effective interactions with second order accuracy. As a point of reference, we consider the free energy in Equation (15) and interpret only the macroion–microion interaction as a perturbation of the microion Hamiltonian that slowly charges the macroions, that is *H_µ_*(*λ*) = *H**_µ_* + *U*_m__+_(*λ*) + *U*_m_*_−_*(*λ*), where the parameter *λ* ∈ [0, 1] denotes the fraction of the full macroion charge.

Within this framework, the free energy of the microions can be formulated as [23]:

(16) $$F_{µ}=F_{\mu }^{\left(0\right)}+\int_{0}^{1}{\langle \frac{\partial }{\;\partial \lambda }\left[{\overset{-}{U}}_{m+}(\lambda )+{\overset{-}{U}}_{m-}(\lambda )\right]\rangle }_{\lambda }d\lambda$$

Here 〈·〉*λ* denotes the ensemble average for a system with partially charged macroions and $F_{\mu }^{\left(0\right)}$ denotes the reference free energy of the microion plasma in the presence of fully penetrable, neutral macroions, given by:

(17) $$F_{\mu }^{\left(0\right)}=-\frac{1}{\beta }\text{ln}\left[\text{tr}\sigma \left(e^{-\beta \overset{-}{H}µ}\right)\right]$$

If we now consider a microion plasma in the presence of neutral macroions and augment the charge adiabatically from zero to full charge, the cross-interaction would correspond to a Hamiltonian *U*_m_*_σ_*(*λ*) = *λ**U*_m_*_σ_*. This simplifies Equation (16) to:

(18) $$\;F_{µ}=F_{\mu }^{\left(0\right)}+\int_{0}^{1}\left({\langle {\overset{-}{U}}_{m+}\rangle }_{\lambda }+{\langle {\overset{-}{U}}_{m-}\rangle }_{\lambda }\right)d\lambda$$

By switching to the Fourier formalism and with the aid of Equations (6) and (7), it is possible to express either of the ensemble averages in terms of densities, the pair interactions, and the background energy:

(19) $$\begin{array}{l} {\langle {\overset{-}{U}}_{m\sigma }\rangle }_{\lambda }=\frac{1}{V}\sum_{k\neq 0}{\hat{\rho }}_{m}(-k){\hat{v}}_{m\sigma }(k){\langle {\hat{\rho }}_{\sigma }(k)\rangle }_{\lambda }\\ +\underset{k\to 0}{\text{lim}}N_{\text{m}}n_{\sigma }[{\hat{v}}_{m\sigma }(k)-Z{\hat{v}}_{-\sigma }(k)] \end{array}$$

Note that the correction terms proportional to *E*b as given in Equation (14) have been cast in a slightly different but identical form. This enables us to demonstrate more clearly that they cancel the divergent terms in the macroion–microion interaction that arise from the Coulomb interaction.

Under the assumption of a linear response of the induced microion density due to the external macroion–microion interaction, as is for instance detailed in Chapter 10 of [23], one finds for ***k*** ≠ 0:

(20) $${\langle {\hat{\rho }}_{\sigma }(k)\rangle }_{\lambda }=\lambda [\chi_{\sigma +}(k)-\chi_{\sigma -}(k)]{\hat{v}}_{m+}(k){\hat{\rho }}_{m}(k)$$

where *χ_µν_*(*k*) with *µ, ν* = *±* are the linear response functions of the two-component microion plasma and we already used that within our model we have *v̂*_m_*_−_* = *−v̂*_m__+_, and in addition *χ_−_*_+_(*k*) = *χ*_+_*_−_*(*k*). As outlined in [20,23] these linear response functions are proportional to the partial structure factors *S_µν_*(*k*). Employing the Ornstein–Zernike relation for mixtures, we can simplify these relations further by expressing the combination of response functions in terms of the microion densities and the inverse Debye screening length *κ* = (4*πn_µ_βq*^2^*/ε*)^1/2^, yielding:

(21a) $$\chi_{++}(k)-\chi_{+-}(k)\;\;=-\frac{\beta_{n+}}{1+\frac{\kappa^{2}}{k^{2}}}$$

(21b) $$\chi_{+-}(k)-\chi_{--}(k)=\frac{\beta_{n-}}{1+\frac{\kappa^{2}}{k^{2}}}$$

Combining Equations (18)–(21) the counterion free energy reads:

(22) $$\begin{array}{l} F_{µ}=F_{\mu }^{\left(0\right)}+\frac{1}{2V}\sum_{k\neq 0}\chi (k){\left[{\hat{v}}_{\text{m}+}(k)\right]}^{2}{\hat{\rho }}_{\text{m}}(-k){\hat{\rho }}_{\text{m}}(k)\\ +\underset{k\to 0}{\text{lim}}N_{\text{m}}(n_{+}-n_{-}){\hat{v}}_{\text{m+}}(k)+2E_{\text{b}} \end{array}$$

where the response function *χ*(*k*) is given by:

(23) $$\begin{array}{l} \chi (k)=\chi_{++}(k)-2\chi_{+-}(k)+\chi_{--}(k)\\ =-\frac{\beta n_{µ}}{1+\frac{\kappa^{2}}{k^{2}}}=-\frac{\varepsilon k^{2}\kappa^{2}}{4\pi q^{2}(k^{2}+\kappa^{2})} \end{array}$$

The form of this free energy expression suggests that one can define an induced interaction *v̂*_ind_(*k*) = *χ*(*k*)[*v̂*_m_*_σ_*(*k*)]^2^ and consequently also an effective interaction *v̂*_eff_ = *v̂*_mm_ + *v̂*_ind_. In doing so, we find that the effective Hamiltonian *H*_eff_ = *H*_m_ + *F_µ_* is given by:

(24) $$H_{\text{eff}}=K_{\text{m}}+\sum_{i<j}^{N_{\text{m}}}v_{\text{eff}}(|R_{i}-R_{j}|)-E_{\text{b}}+E_{0}$$

where *K*_m_ is the macroion kinetic energy, and by *E*_0_ we denote the so-called volume energy, which reads:

(25) $$\begin{array}{l} E_{0}=F_{\mu }^{\left(0\right)}+\frac{N_{\text{m}}}{2}\underset{r\to 0}{\text{lim}}v_{\text{ind}}(r)+\frac{N_{\text{m}}n_{\text{m}}}{2}\times \\ \underset{k\to 0}{\text{lim}}\{-{\hat{v}}_{\text{ind}}(k)+2Z[{\hat{v}}_{m+}(k)+Z{\hat{v}}_{++}(k)]\} \end{array}$$

where we used charge neutrality, Equation (12) and the inverse Fourier transformation. The volume energy *E*_0_ is a natural consequence of the reduction procedure from a complex mixture to an effective one-component system. Although it does not depend explicitly on the macroion coordinates, it can still have a significant contribution to the free energy due to its dependence on the overall macroions density.

This completes the one-component reduction and linear response approximation of our model microgel solution. The next task is that of explicitly calculating the microion density profiles $\langle \rho_{\pm }(r)\rangle$ around a single macroion placed, for convenience, at the coordinate origin and the effective pair interaction energy *v*_eff_(*r*) between two macroions.

## 3. Microion Densities and Effective Pair Interaction

We will assume that our multilayered microgel particles in first approximation consist of a spherically symmetric charge distribution that can be accurately described in terms of *l* layers of piecewise constant charge densities *ϱ̃_i_*. Each of these densities can be positive, negative or even zero, and the corresponding *i*th layer is a spherical shell of thickness *d_i_* = *a_i_* *−* *a_i−_*_1_. Here the *a_i_* denote the radii of the spheres bounding the shells. In the case *i* = 1 we write the innermost domain just as a spherical core of charge density *ϱ̃*_1_ with radius *a*_1_. Representing the layers by linear combinations of Heaviside functions, we obtain the charge distribution of a macroion placed at the origin as:

(26) $$\varrho (x)=\sum_{i=1}^{l}\varrho_{i}\Theta (a_{i}-|x|)$$

where *ϱ_l_* = *ϱ̃_l_* and *ϱ_i_* = *ϱ̃_i_* *−* *ϱ̃_i_*_+1_ for 1 *≤* *i < l*. This representation of the layered structure as a superposition of homogeneously charged spheres is strictly speaking not required, but is more convenient than treatment by means of shells.

The total direct interaction between a pair of layered microgel particles can be calculated by means of a six-dimensional integral over the charge densities:

(27) $$\begin{array}{l} v_{\text{mm}}(r)=\frac{1}{\varepsilon }\iint \frac{\varrho (x)\varrho (y-r)}{\left|x-y\right|}d^{3}xd^{3}y\\ =\frac{1}{\varepsilon }\sum_{i=1}^{l}\varrho_{i}^{2}\Phi_{ii}(r)+\frac{2}{\varepsilon }\sum_{j<i}^{l}\varrho_{i}\varrho_{j}\Phi_{ij}(r) \end{array}$$

where:

(28) $$\Phi_{ij}(r):=\iint \frac{\Theta (a_{i}-|x|)\Theta (a_{j}-|y-r|)}{\left|x-y\right|}d^{3}xd^{3}y$$

and ***r*** denotes the center–center separation of the two macroions. For non-overlapping macroions, Φ*_ij_* is clearly the Coulomb potential. The situation for overlapping macroions can be reduced to a two-dimensional integral employing cylindrical symmetry, resulting in:

(29) $$\Phi_{ij}(r)=2\pi \int_{0}^{a_{j}}\int_{0}^{\pi }\Phi_{i}(|x+r|)x^{2}sin\vartheta d\vartheta dx$$

with Φ*_i_* being the potential of a charged sphere of radius *a_i_*. Note that Φ*_ij_* is actually a radially symmetric function, and hence this expression can be shown, under the condition *a_j_* *≤* *a_i_*, to yield:

(30) $$\begin{array}{l} \Phi_{ij}(r)=\frac{16\pi^{2}}{9}\{\begin{array}{ll} \frac{a_{j}^{3}}{10}\left(15a_{i}^{2}-3a_{j}^{2}-5r^{2}\right) & 0\leq r<a_{i}-a_{j}\\ \begin{array}{l} \frac{a_{i}^{3}a_{j}^{3}}{r}\text{+}\frac{1}{160r}{\left(a_{i}+a_{j}-r\right)}^{4}\\ \times \left[5\left(a_{i}^{2}-4a_{i}a_{j}+a_{j}^{2}\right)-4(a_{i}+a_{j})r-r^{2}\right] \end{array} & a_{i}-a_{j}\leq r<a_{i}+a_{j}\\ \frac{a_{i}^{3}a_{j}^{3}}{r} & a_{i}+a_{j}\leq r \end{array} \end{array}$$

Having derived the macroion pair potential, we now focus on the average microion densities. From Equations (20), (21) and (23), we obtain for ***k*** *≠* 0:

(31) 〈*ρ̂_±_*(***k***)〉 = *±x_±_χ*(*k*)*v̂*_m__+_(*k*)*ρ̂*_m_(***k***)

Here *x_±_* = *n_±_/n_µ_* denote the fraction of positive and negative microions, respectively, and:

(32) $$\begin{array}{l} {\hat{v}}_{\text{m+}}(k)=\frac{q}{\varepsilon }\sum_{i=1}^{l}\varrho_{i}{\hat{\Phi }}_{i}(k)\\ =-\frac{16\pi^{2}q}{\varepsilon k^{4}}\sum_{i=1}^{l}\alpha_{i}\varrho_{i}\left[\text{cos}(ka_{i})-\frac{\text{sin}(ka_{i})}{ka_{i}}\right]\; \end{array}$$

is the Fourier transform of the macroion–microion pair potential. Combining Equations (31) and (32) yields:

(33) $$\begin{array}{l} \langle {\hat{\rho }}_{\pm }(k)\rangle =\pm x_{\pm }\frac{4\pi }{q}\sum_{i=1}^{l}\varrho_{i}a_{i}\frac{\kappa^{2}}{k^{2}(k^{2}\;+\kappa^{2})}\;\\ \times \left[\text{cos}(ka_{i})-\frac{\text{sin}(ka_{i})}{ka_{i}}\right]\sum_{j=1}^{N_{\text{m}}}e^{ik\cdot R_{j}}\\ +\delta (k)[N_{\pm }\mp x_{\pm }ZN_{\text{m}}] \end{array}$$

where the second term containing the Dirac delta function *δ*(***k***) has been added to ensure the correct behavior in the small wave vector limit, *i.e.*, $\langle {\hat{\rho }}_{\pm }(k)\rangle =\;N_{\pm }$ for *k* *→* 0. The microion densityprofiles around a single macroion can now be obtained by means of the inverse Fourier transformation. The particular form of these expressions implies that 〈 *x_−_ρ̂*_+_(***k***) + *x*_+_*ρ̂_−_*(***k***)〉 = 2*x*_+_*x_−_N_µ_δ*(***k***) and hence that 〈*x−ρ̂*_+_(***r***) + *x*_+_*ρ̂_−_*(***r***)〉 is a constant and can be written in the following form:

(34) $$\langle \rho_{\pm }(r;\{R\})\rangle =2\frac{n_{+}n_{-}}{n_{µ}}\pm x_{\pm }\sum_{j=1}^{N_{\text{m}}}\xi (|r-R_{j}|)$$

where we explicitly have written them as a function of the particular macroion configuration, denoted by {***R***}, and we introduced the charge density response to the macroions *ξ*(*r*) given by:

(35) $$\begin{array}{l} \xi (r)=-\frac{1}{qr}\{\sum_{i=m+1}^{l}\varrho_{i}a_{i}\\ \times \left[\frac{r}{a_{i}}-\left(1+\frac{1}{\kappa a_{i}}\right)e^{-\kappa a_{i}}\text{sinh}(\kappa r)\right]\\ +\sum_{i=1}^{m}\varrho_{i}a_{i}e^{-\kappa r}\left(\text{cosh}(\kappa a_{i})-\frac{\text{sinh}(\kappa a_{i})}{\kappa a_{i}})\right\} \end{array}$$

Here *m* is the number corresponding to the layer in which the distance *r* is found, *i.e.*, *a_m_* *≤* *r < a_m_*_+1_, with the formal additions of *a*_0_ = 0 and *a_l_*_+1_ = *∞*. Also note that *ξ*(*r*) is a density deviation and as such is not required to be strictly positive. In fact it can obtain negative values due to its dependence on the charge densities *ϱ_i_* describing the macroion structure. However, when it is integrated over the available volume it gives exactly the valence *Z* of the macroions and therefore its volume averaged value is positive.

Using the previously identified induced interaction *v̂*_ind_(*k*) = *χ*(*k*)[*v̂*_m__+_(*k*)]^2^, the effective interaction *v*_eff_ = *v*_mm_ + *v*_ind_ can now be derived. To this end, Equations (23) and (32) need to be combined with the Fourier transform of *v*_mm_(*r*), which results in:

(36) $$\begin{array}{l} {\hat{v}}_{\text{eff}}(k)=\frac{64\pi^{3}}{\varepsilon k^{4}}\frac{1}{k^{2}+\kappa^{2}}\sum_{i,j=1}^{n}\varrho_{i}\varrho_{j}a_{i}a_{j}\\ \times \left(\text{cos}(ka_{i})-\frac{\text{sin}(ka_{i})}{ka_{i}}\right)\\ \times \left(\text{cos}(ka_{j})-\frac{\text{sin}(ka_{j})}{ka_{j}}\right) \end{array}$$

The only remaining task is to apply the inverse Fourier transform to *v̂*_eff_(*k*) in order to obtain the effective interaction between two macroions, which has the following form:

(37) $$v_{\text{eff}}(r)=\frac{32\pi }{\varepsilon r}\sum_{i,j=1}^{l}\varrho_{i}\varrho_{j}a_{i}a_{j}I_{ij}$$

where for *I_ij_*, just as in Equation (30), three cases need to be distinguished. For 0 *≤* *r < a_i_* *−* *a_j_*, the case where the smaller sphere with radius *a_j_* is completely contained within the larger one with radius *a_i_*, it reads:

(38) $$I_{ij}=\frac{\pi }{6a_{i}\kappa^{5}}\left[a_{j}^{2}\kappa^{3}r-3(a_{i}\kappa +1)e^{-\kappa a_{i}}\text{sinh}(\kappa r)\times \left(\text{cosh}(\kappa a_{j})-\frac{\text{sinh}(\kappa a_{j})}{\kappa a_{j}}\right)\right]$$

In the intermediate case *a_i_−* *a_j_≤* *r < a_i_* + *a_j_*, when both spheres are intersecting, we find:

(39) $$\begin{array}{l} I_{ij}=\frac{\pi }{96a_{i}a_{j}\kappa^{6}}[\kappa^{4}r^{4}-6\left(\kappa^{2}\left[a_{i}^{2}+a_{j}^{2}\right]-2\right)\left(\kappa^{2}r^{2}+2\right)\\ \begin{array}{l} +8\kappa^{4}(a_{i}^{3}+a_{j}^{3})r-3\kappa^{4}{\left(a_{i}^{2}-a_{j}^{2}\right)}^{2}\\ +12(\kappa a_{i}+1)(\kappa a_{j}-1)e^{-\kappa (a_{i}-a_{j})}e^{-\kappa r}\\ +12(\kappa a_{i}-1)(\kappa a_{j}+1)e^{-\kappa (a_{j}-a_{i})}e^{-\kappa r} \end{array}\\ -24(\kappa a_{i}+1)(\kappa a_{j}+1)e^{-\kappa (a_{i}+a_{j})}\text{sinh}(\kappa r)] \end{array}$$

Finally, in the case *ai* + *aj* *≤* *r* for which both spheres are fully separated, we obtain:

(40) $$\begin{array}{l} \;I_{ij}=\;\frac{\pi }{2\kappa^{4}}e^{-\kappa r}\left(\text{cosh}(\kappa a_{i})-\frac{\text{sinh}(\kappa a_{i})}{\kappa a_{i}}\right)\\ \times \left(\text{cosh}(\kappa a_{j})-\frac{\text{sinh}(\kappa a_{j})}{\kappa a_{j}}\right) \end{array}$$

In the particular case of non-overlapping macroions with a homogeneous charge density, the effective interaction in Equation (37) would only contain a single term of the type in Equation (40) and hence the distance dependence follows the expected Yukawa behavior exp(*−κr*)*/εr*. In the limit *κa* *→* 0 the effective charge corresponds to the usual result that one finds from the DLVO theory, *i.e.*, *−Zq*exp(*κa*)/(1 + *κa*). In general however, the effective charge will only be a fraction of that result, which decreases monotonously as function of *κa*, *i.e.*, on both increasing the overall microion concentration, as expected, and on increasing the macroion size. The latter is because here the microions are not restricted to remain outside the macroion but can actually penetrate into the macroion and therefore screen the bare charge even more effectively.

## 4. Application to Core–Shell Microgels

In order to illustrate the results for inhomogeneous microgels we derived in the previous sections, we apply it to the limited case of core–shell particles by setting *l* = 2. We assume the macroions to have a radius *a*_2_ = *a* and that the core size is given by the fraction *a*_1_ = *αa* with *α* *∈* [0, 1]. This implies that the shell has a width *d*_2_ = (1 *−* *α*)*a*. Having the total charge of the gel particle already specified to be *−Zq*, the total charge in core and shell can be described by means of an unrestricted dimensionless quantity *ζ* to yield *Q*_1_ = *−*(1 *−* *ζ*)*Zq* and *Q*_2_ = *−ζZq* respectively. Therefore, the charge density of the macroion introduced in Equation (26) reads:

(41) $$\varrho (x)=\varrho_{2}\Theta (a_{2}-|x|)+\varrho_{1}\Theta (a_{1}-|x|)$$

with:

(42a) $$\varrho_{1}=-\frac{3Zq(1-\alpha^{3}-\zeta )}{4\pi a^{3}\alpha^{3}(1-\alpha^{3})}$$

(42b) $$\varrho 2=-\frac{3Zq\zeta }{4\pi a^{3}(1-\alpha^{3})}.$$

As an example of core–shell microgels, we fix the total charge by setting *Z* = 100, the radius to *a* = 50 nm, and target monovalent microions as already chosen, *i.e.*, *z* = 1. We select two particular and representative examples of these class of particles and take in either case the radius of the core to be half of the total radius by setting *α* = 1/2. The first example is characterized by *ζ* = 1/3 and results in a particle with a core that contains twice as much charge as the shell. For the second case we take *ζ* = 4/3, which results in a particle with a positive core and a negative shell, where the total charge contained in the latter is four times larger and of opposite sign. In both cases the absolute charge density within the shell is lower than that of the core. For the solvent we assume water at room temperature, which within the model is characterized by the relative permittivity *E* = 80.

Note that these particular choices are made in order to illustrate the method and highlight the effects of radial inhomogeneous charge distributions on both the counterion distributions and the effective interactions. The first case corresponds to the more commonly expected charge distribution in microgels, with a less densely charged or even neutral core surrounded by a charged shell. The oppositely charged core and shell in the second example might be somewhat unexpected. However, it is possible to make dendrimers with this type of architecture [26].

Using the formalism derived in the previous sections, the radial density profiles 〈*ρ_±_*(*r*)〉 of microions and the effective pair interaction *βv*_eff_(*r*) between the microgel particles have been computed for both cases. The results are shown in and for four different salt concentrations specified by the value *κa*. As a reference, the results for homogeneously charged particles with the same overall charge and size have been included as well and are indicated by the dashed curves.

**Figure 1:**
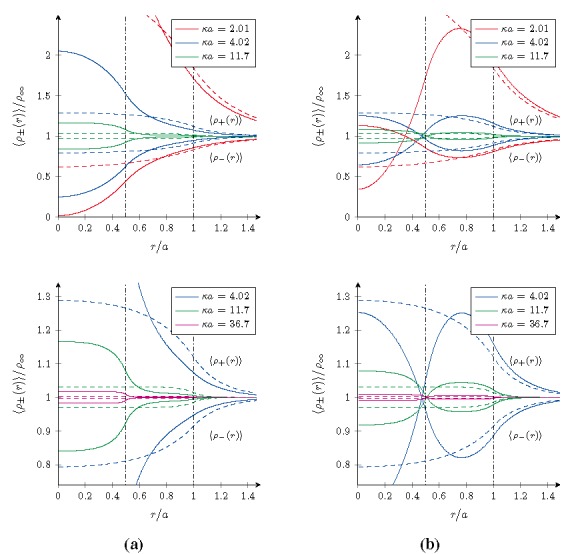
Radial density profiles 〈*ρ_±_*(*r*)〉/*ρ_∞_* of microions near core–shell microgels for different salt concentrations at room temperature. (**a**) A negative core and shell for *ζ* = 1/3; and (**b**) a positive core and negative shell for *ζ* = 4/3. The dashed curves are the reference results for a homogeneously charged microgel with the same overall charge and size, and the vertical lines form the borders between different types of overlapping. The bottom graphs show zoomed versions of the top graphs in order to focus on the higher salt concentrations.

The microion density profiles around a single gel particles shown in Figure 1 are normalized with *ρ_∞_* *≡* 2*n*_+_*n_−_*/*n_µ_*, the bulk density of microions far away from the macroion. This enables us to focus on the structure in the profiles, rather than their absolute values. Within these profiles three different ranges can be identified, which reflect the internal two-layered structure of the gel particle chosen here for illustrative purposes, *i.e.*, inside the core *r* *≤* 0.5*a*, within the shell 0.5*a* *≤* *r* *≤* *a*, and outside the particle *r > a*. In the outer region, one finds the exponential decrease of the positive microion densities *ρ*_+_(*r*) and the corresponding increase of the negative microion densities *ρ_−_*(*r*), both ultimately converging to *ρ_∞_*. This behavior reflects the recovering of the Yukawa interaction and is due to the balancing of increased entropy from microions that are found in the solvent versus the energy cost of having a non-neutral charge density distribution.

In the case *ζ* = 1/3 of the negative core and shell, the densities $\langle \rho_{+}(r)\rangle$ and $\langle \rho_{-}(r)\rangle$ in this outer range are a bit lower and higher respectively, with respect to those for a homogeneously charged particle. This effect can be understood in the light of the higher charge density found within the core that will bind more counterions, which therefore are depleted more from the outer regions. Hence the opposite trend for these densities in the innermost region and cross-over in the intermediate range. Obviously, the effect itself is diminished on increasing the salt concentration.

**Figure 2:**
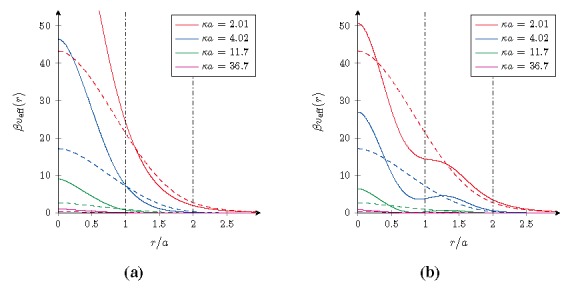
Effective pair interactions *v*_eff_(*r*)/*k*_B_*T* at room temperature *T* between core–shell microgels for different salt concentrations. (**a**) A negative core and shell for *ζ* = 1/3; and (**b**) a positive core and negative shell for *ζ* = 4/3. The dashed curves are the reference results for homogeneously charged microgels with the same overall charge and size, and the vertical lines form the borders between different types of overlapping.

For the other case *ζ* = 4/3 with a positive core and negative shell, a similar argument can be used. However, the local charge density in the shell is now higher than that found in a homogeneously charged macroion, and consequently also the number of counterions found here is higher with respect to the reference system. Contrary to the case *ζ* = 1/3, the density of positive microions is not larger than the bulk value in the whole range, but becomes smaller in the core as they are depleted from the now likewise positively charged core. The cross-over behavior is again in the intermediate range and the addition of salt desensitizes the density profiles to the internal structure of the microgel.

Figure 2 displays the effective pair interaction in Equation (37) for the two particular cases we consider here. As was the case for the density profiles, three different ranges can be also for the effective interaction identified, the separation of which is marked by vertical lines. For large distance *r >* 2*a* the gel particles are fully separated and essentially interact by means of an effective charge in a screening solvent. The magnitude and sign of this effective charge is determined by the inhomogeneous charge within the particles. In the intermediate range *a* ≲ *r <* 2*a*, there is partial overlapping of the shells, and in the shortest distance range *r* ≲ *a* even the cores start to overlap mutually.

In the case *ζ* = 1/3, the charge density in the shell is lower than in the core, which weakens the strength of the interaction in the intermediate range with respect to that of a homogeneous particle with the same total charge and size. Only when the higher charge density cores get close enough, the interactions increase to be stronger and settles at values more than twice as large as for the homogeneously charged macroion. In contrast, for the case of *ζ* = 4/3 where the shell has a higher charge density, the pair interaction is enhanced in the intermediate range. But now, on approaching of the cores, the repulsive force decreases and even becomes attractive as can be seen from the flattening-off and decline in the effective interaction upon increasing the salt concentration. This is merely a consequence of the overlapping between the oppositely charged cores and shells, and this attractive behavior ceases to exist for sufficient overlap between the cores of both gel particles. Also in this case the effective interaction is higher than that for the homogeneously charged particle, but not as high as for the previous case. In the former case an increase in the salt concentration only results in a reduction of the effective interaction potential by means of a more efficient screening, yet in the latter case it can actually be used to induce a small and relatively weak attractive range of interactions. However, it should be realized that such behavior does require a substantial overlap between the macroions and that their steric interactions, which have not been taken into account, will be most likely sufficient to remove this effect.

## Conclusions

We have set up a theoretical framework to describe systems of inhomogeneous gel particles. We made a one-component reduction of the complex system of gel particles, counterions, solvent, and added salt to an effective description of the macromolecules only. This is combined with the application of linear response theory to implement the electrostatic governed interactions of the microscopic level. The gel particles have been assumed to be radial symmetric and static in structure so that they can be considered to be accurately described as a spherical multilayered object. These macroions are also assumed to be fully penetrable with respect to each other, *i.e.*, steric interaction are completely neglected and the interaction is driven solely by the Coulomb interactions. This is a simplification and in general will not be valid, but still results in a qualitatively correct description that ultimately should be tested in a more rigorous fashion by, for instance, computer simulations. In the case of charged dendrimers [22], where fully intertwined conformations are not hindered by cross-links as found in microgels, microion density profiles are observed that exhibit the same plateau-like structures seen in the case studies presented here. The framework is fully general and can be readily applied to any spherical multilayered structure and has here only been illustrated by considering two particular cases of core–shell microgels. One of the chosen microgels was formed by a core and shell with only different negative charge densities. Such an inhomogeneity will affect the interactions and microion densities mainly in the short range. The other example, where a positively charged core is surrounded by a negatively charged shell, reveals the more interesting aspects of charge inhomogeneities in microgel particles, as it shows the potential of inducing a range of, albeit weak, attractions between macroions that have an overall likewise total charge. Such behavior can be tuned within the framework provided here by modifying the relative size of core and shell, the local charge densities, and possibly the addition of extra layers. Whether such interaction could be made strong enough to overcome the steric interactions, however, remains an open problem and is beyond the scope of this work.
